# Graphene Oxide-Gold Star Construct on Triangular Electrodes for Alzheimer's Disease Identification

**DOI:** 10.1155/2021/6661799

**Published:** 2021-02-22

**Authors:** Wenlong Chang, Jing Zhao, Lu Liu, Xiaoming Xing, Chao Zhang, Huihong Meng, Subash C. B. Gopinath, Yonggang Liu

**Affiliations:** ^1^Endocrine Laboratory, Baoding No.1 Central Hospital, Baoding, Hebei 071000, China; ^2^Department of First Neurology, Baoding No.1 Central Hospital, Baoding, Hebei 071000, China; ^3^Department of Clinical Psychology, Baoding No.1 Central Hospital, Baoding, Hebei 071000, China; ^4^Faculty of Chemical Engineering Technology, Universiti Malaysia Perlis (UniMAP), Arau 02600, Perlis, Malaysia; ^5^Institute of Nano Electronic Engineering, Universiti Malaysia Perlis (UniMAP), Kangar 01000, Perlis, Malaysia

## Abstract

Nanotechnology is playing a major role in the field of medical diagnosis, in particular with the biosensor and bioimaging. It improves the performance of the desired system dramatically by displaying higher selectivity and sensitivity. Carbon nanomaterial, gold nanostructure, magnetite nanoparticle, and silica substrate are the most popular nanomaterials greatly contributed to make the affordable and effective biosensor at low-cost. This research work is introducing a new sensing strategy with graphene oxide-constructed triangular electrodes to diagnose Alzheimer's disease (AD). MicroRNA-137 (miRNA-137) was found as a suitable biomarker for AD, and the sensing method was established here to detect miRNA-137 on the complementary sequence. To enhance the immobilization of capture miRNA-137, gold nanostar (GNS) was conjugated with capture miRNA and immobilized on the GO-modified surface through an amine linker. This immobilization process enhanced the hybridization of the target and reaches the detection limit at 10 fM with the sensitivity of 1 fM on the linear curve with a regression coefficient of 0.9038. Further control sequences of miRNA-21 and single and triple base mismatched miRNA-137 did not show a significant response in current changes, indicating the specific miRNA-137 detection for diagnosing AD.

## 1. Introduction

Nanomaterial-based biosensors can remarkably improve the specificity and sensitivity of the interactive biomolecules on the sensing surface, lead to the applications in pathogenic diagnosis, recognition of biomolecules, and environmental monitoring [1–5]. Nanomaterials including carbon-based material, silver nanoparticle, gold nanoparticle, magnetic nanoparticle, and silica nanoparticle have been found greatly improving biomolecular detection and help to reach the lower detections of the desired target molecule [6–11]. Graphene is an attractive material with one atom thick having a honeycomb structure with the sp2-bonded carbon. Recently, graphene derivatives have been attracted to construct the biosensor due to their excellent biocompatibility, mechanical strength, high thermal conductivity, higher elasticity, and property [12, 13]. In particular, electrochemical and electrical sensors are more popular due to the physiochemical properties of graphene [14, 15]. Graphene-modified sensing surfaces yield the sensitive and more stable sensing method for the detection of various blood-based biomarkers to identify the pathogens, viruses, and also diseases such as cancer and diabetes [16–18]. This research is constructing a microdevice with triangular electrodes using graphene oxide (GO) to diagnose Alzheimer's disease biomarker, microRNA-137 (miRNA-137).

Alzheimer's disease (AD) is the progressive disorder, causing the cells of the brain to die or degenerate. AD is the common cause of dementia in humans and results in declining thinking, disturbing social skill, and behavioral changes [19]. AD is in the advanced stages causing serious memory impairment and losing the ability to perform everyday task. Approximately 37 million people are suffering from AD worldwide. Until now, there is no complete strategy to cure AD, and there is no concrete method to diagnose AD except the strategy such as brain postmortem examination. So that, developing a point-of-care diagnosing method is mandatory for the diagnosis of AD [20]. Herein, a novel graphene-modified triangular electrode to diagnose AD assisted by microRNA-137 (miRNA 137) was introduced.

MicroRNA (miRNA) is a short, noncoding RNA that can stimulate either mRNA degradation or its translational repression, which helps to regulate the gene expression. miRNA can bind specifically with its target mRNA in order to inhibit or decrease the expression. Recently, miRNA is more popularized and developing rapidly; in particular, the circulating miRNAs were found as the reliable modern biomarker for disease identification [21]. miRNA-137, containing 23 nucleotides, is found as the noninvasive reliable biomarker for AD [22]. In this study, miRNA-137 was identified by the complementary of miRNA-137 (comp-miRNA-137) on the graphene-modified sensing surface as the probe. To enhance the immobilization of comp-miRNA-137, gold nanostar (GNS) was utilized. Probe-conjugated gold nanomaterial improves the stability of the molecule and increases the numbers of probe attachment on the sensing surface [1, 23, 24]. To demonstrate the AD sensing here, comp-miRNA was conjugated. GNS was immobilized on the sensing surface by using the amine as the linker and then identified miRNA-137 specifically. This study has brought the novelty by a new composite of graphene and gold star complex, as this combination has not been revealed earlier. It deviates from the previously reported conjugations of other gold structures to GO by providing an extended surface area. Furthermore, the chemical functionalization followed made a strong attachment of gold star-GO complex on the sensing surface desired in this research. Inclusion of PEG-COOH promotes the nonfouling and high-performance of the sensor.

## 2. Materials and Methods

### 2.1. Oligos and Chemicals

3-Aminopropyltrimethoxysilane (APTMS), phosphate buffer saline (PBS), deoxyadenosine triphosphate (dATP), potassium hydroxide (KOH), ethanolamine, gold nanostar (GNS), and graphene oxide (GO) were purchased from Sigma-Aldrich, USA. Silica wafer, standard cleaning solutions 1 and 2 (RCA1 and RCA2), positive (PRI 2000 A) and negative (NR7 6000PY) photoresists, and resist developer (RD) were ordered from Futurrex Inc., Franklin, USA. The following oligonucleotides were synthesized commercially from the local supplier [22]. Probe miRNA-137: 5′-SH-UUAUUGCUUAAGAAUACGCGUAG-3′; comp-miRNA-137: 5′-CUACGCGUAUUCUUAAGCAAUAA-3′; single base-mismatched miRNA-137: 5′-UUAUUGCUUAACAAUACGCGUAG-3′; triple base mismatched miRNA-137: 5′-UUAUUGCUUATCTAUACGCGUAG-3′. Graphene oxide (GO) was prepared by the method outlined by Gopinath et al. [25].

### 2.2. Triangular Electrodes Sensor Design and Fabrication

The pattern of the triangular electrodes sensor surface was designed by AutoCAD software with the gap at the junction with ∼20 *μ*m. A conventional technique of lithography was utilized for the fabrication of the sensor. At first, the base substrate of silica wafer was washed with cleaning solutions RCA1 and RCA2 to remove the impure substances. On the washed surface, wet oxidation was performed to grow the oxide layer, and then, the aluminum (Al) layer was deposited and patterned by using the technique of reactive ion etching. Finally, the fabricated sensor surface was washed with acetone and distilled water and dried for further surface modifications.

### 2.3. Conjugation of Comp-miRNA-137 and GNS

Conjugation of probe with GNS was performed using the procedure by Hartati et al. [26]. Briefly, 100 *μ*M of GNS was incubated with dATP with 100 mM in the mole ratio of 1 : 300, and this mixture was kept for 15 min at room temperature. After that, the solution was diluted with PBS (10 mM, pH 7.4), and then, 0.1 mM of comp-miRNA-137 (mole ratio of 30 : 1) was added by heating for 3 h at 60°C to reach the equilibrium. This solution was separated by centrifugation at the speed of 1000 rpm for 15 min and washing. The precipitation was dissolved in PBS and kept at the refrigerator for further use.

### 2.4. Modification on the Triangular Electrode Sensor Surface

To construct GO on the sensor, 1 mg of GO was dispersed in diluted APTMS (0.25%) and rest at room temperature (RT) for 1 h. In parallel, a sensor substrate was treated with 1% of diluted KOH to crate the hydroxyl group. After that, GO dispersed in APTMS was introduced on the KOH treated surface and then immediately dropped comp-miRNA-GNS to achieve the final capture miRNA modified surface to determine miRNA-137.

### 2.5. Hybridization of miRNA-137 on the Triangular Electrodes Surface

Hybridization of miRNA-137 with its complementary sequence immobilized sensor surface was conducted after the surface was blocked by the blocking agent, PEG-COOH. About 5 *μ*l of miRNA at the concentration of 100 pM was initially placed on the comp-miRNA-137-GNS modified sensor surface and rested for 30 min at RT. After washing the surface with PBS buffer (10 mM; pH 7.4), the current response was measured at the voltage from 0 to 2. To determine the detection limit, miRNA-137 was diluted from 1 fM to 10 pM, and the similar hybridization experiment was conducted to calculate the current differences for each concentration. Difference in current was plotted in an excel sheet, and the limit of detection with its regression coefficient value was calculated.

### 2.6. Selective and Specific miRNA-137 Detection

Specific miRNA-137 detection was identified with control miRNA-21 sequence and single and triple base mismatched miRNA-137 sequences. Instead of miRNA-137, these three sequences were placed individually on the comp-miR-137 modified sensor surface, and the current responses to find the specific detection of miRNA-137 were recorded. Furthermore, selective miRNA-137 detection experiment was conducted by mixing miRNA-137 (1 fM) with 100 pM of miRNA-21, 100 pM of miRNA-155, and 1 : 100 diluted human serum. This complex was dropped on the capture probe modified sensor surface. Then, the surface was washed by PBS buffer to remove the unattached oligos, and the current response was recorded.

## 3. Results and Discussion

**Figure 1:**
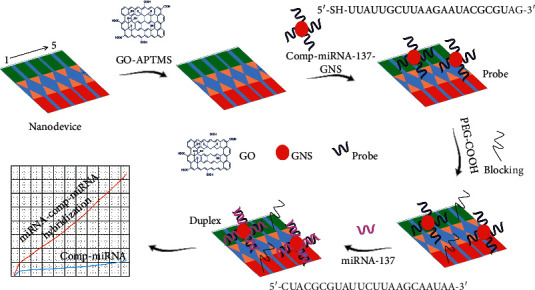
Schematic illustration of miRNA-137 determination. Sensing substrate was modified into APTMS-GO, and then, gold nanostar-conjugated complementary of miRNA-137 (GNS-probe) was attached and then detected by miRNA-137 complementation. A set of five devices is shown.

Diagnosing Alzheimer's disease (AD) with a suitable biomarker is greatly important to provide the right medications and improve the patient lifestyle. MiRNA-137 was found as the biomarker to diagnose AD patients and their condition. This research work focuses to identify the AD biomarker, miRNA-137, on the graphene-constructed triangular electrodes sensor surface.  Figure 1 represents the schematic illustration of miRNA-137 determination on the sensor surface. To layer the GO on the sensing surface, APTMS was used as the linker as it reacts the oxide materials. The sensing surface was originally made with hydroxyl groups to capture amine on APTMS. Furthermore, the embedding of gold star was made possible by anionic and cationic reactions between gold and APTMS. Before attaching gold star, it was reacted with thiolated-probe molecule (complementary of miRNA-137). As widely agreed, the thiol group can make a strong bonding with the gold surface, and in the current case, higher numbers of probe have been immobilized on the gold star due to the larger surface compared to the spherical particles. The available space after attaching probe reacts with the amine of APTMS on the sensing surface.

Probe immobilization on the sensing surfaces is playing the major role for improving the sensitivity. Probe can align on the sensing surface through various materials, including polyethylene glycol (PEG) and nanoparticles. Nanoparticle-conjugated probe improved the stability of the probe and encourages more probes attachment on the sensing surface. In particular, gold nanomaterials proved to lower the detection limit of the target with various sensors [27]. Gold nanoparticles can easily attach with biomolecules such as antibody, peptide, DNA, RNA, and protein through the thiol linker and immobilized on the sensing surface [28]. Herein, GNS-conjugated probe improved the surface orientation and increased the numbers of probe attachment on the triangular electrode sensor surface. On these probes-modified surfaces, target miRNA-137 sequence was detected through hybridization.

### 3.1. GNS-Probe Immobilization on the GO-Modified Surface

**Figure 2:**
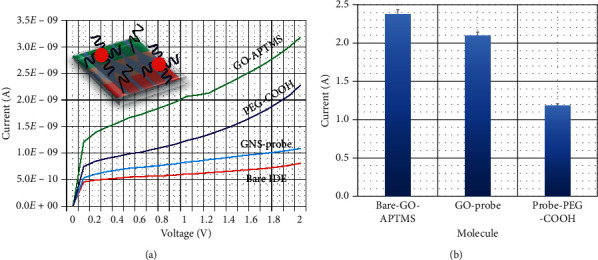
(a) Process and current response of GNS-probe immobilization. After probe immobilization, clear changes in current were noted. Coloured lines are GO-APTMS (green), PEG-COOH (blue), GNS-probe (sky blue), and bare IDE (red). (b) Difference in current change GNS-probe immobilization. Probe attachment shows the highest difference in current.

The GNS-probe was attached on the GO-modified sensor surface through the APTMS linker.  Figure 2(a) shows the process and current response of GNS-probe immobilization on the sensor surface. As described in the figure, the KOH-treated surface shows the current as 8.06*E*−10 A; after modifying the surface with GO-APTMS, current response was increased to 3.18*E*−09 A. This increment of current confirms the construction of GO on the sensor surface. After that, GNS-probe was introduced on the surface, and the current level was changed to 1.09*E*−09 A. This change was due to the attachment of GNS with the GO-APTMS surface. Finally, when the blocking agent PEG-COOH was dropped, the current level was increased to 2.27*E*−09 A. PEG-based polymers reduce the nonspecific binding of biomolecules on the sensing surfaces and reduce the signal-to-noise ratio. Moreover, PEG polymers give the proper orientation of probe attachment on the sensing surface, which attracts higher number of target molecule and reaches the lower detection limit. As shown in Figure 2(b), the difference in current after introducing a PEG-COOH was 1.18*E*−09 A. High difference in current was noted when GNS-probe introduced on the surface confirms the higher number of probes was attached on the GO-modified surface. This comp-miRNA constructed surface was utilized to determine the level of miRNA-137. This increased current confirms the hybridization of miRNA-137 with its immobilized complementary probe sequence.

### 3.2. Hybridization of miRNA-137 with Comp-miRNA-137

**Figure 3:**
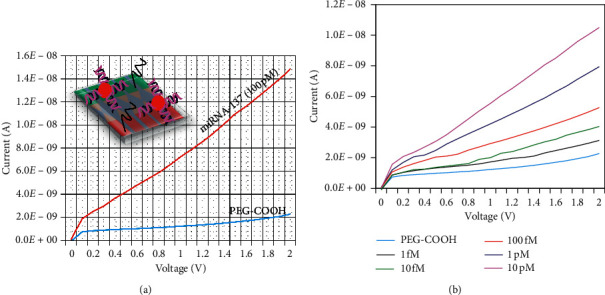
(a) Determination of miRNA-137 on the probe-GNS modified surface. Drastic increment of current was noted after hybridization (100 pM; red line); PEG-COOH is indicated by sky blue line. (b) Different miRNA-137 hybridization levels. 1 fM–10 pM concentrations with constant comp-miRNA-137 on the sensing surfaces. Current increment was noted with all miRNA-137 concentrations. Coloured lines are PEG-COOH (sky blue), 1 fM (black), 10 fM (green), 100 fM (red), 1 pM (blue), and 10 pM (pink).

After blocking the surface with PEG-COOH, the target miRNA-137 at 100 pM was dropped on the surface, and the current was drastically increased to 1.48*E*−08 A. The difference in current was noted as 1.05*E*−08 A (Figure 3(a)). This big change in current was noted due to the higher numbers of miRNA-137 that can hybridize its complementary sequence. The biomolecular interaction on the sensing surface mainly depends on the affinity of the biomolecules and the immobilized probe molecules on the sensing surface. In this research, GNS-conjugated complementary sequence increases the numbers on the sensing surfaces and attracted higher number of target miR-137. This result confirms the hybridization process on the triangular electrode sensing surfaces.

### 3.3. Limit of miRNA-137 Detection

**Figure 4:**
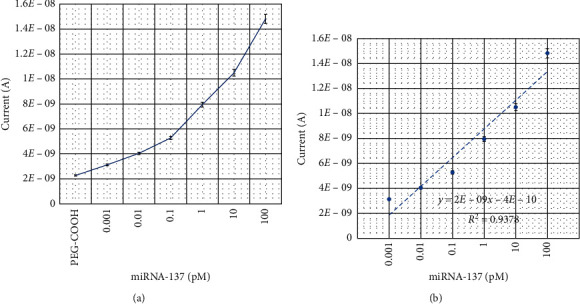
(a) Current response of miRNA-137 hybridization. With increasing target miRNA-137 concentrations, current responses increased gradually. (b) Difference in current response hybridization. miRNA-137 and its complementations are plotted. Detection limit was calculated as 10 fM.

Detection limit of miRNA-137 was determined by conducting the experiment with different miRNA-137 (1 fM–10 pM) hybridization levels with constant comp-miRNA-137 on the sensing surfaces. As shown in Figure 3(b), 1 fM of miRNA-137 shows the current response as 3.13*E*−09 A. Clear changes in current was noted (black line), after adding 1 fM of target sequence. Furthermore, increasing the concentration to 10 fM, 100 fM, 1 pM, and 10 pM, the current levels increased to 4.04*E*−09, 5.27*E*−09, 7.94*E*−09, and 1.05*E*−08 A, respectively. It was clearly noted that with increasing target miRNA-137 concentration, the current response was also increased gradually (Figure 4(a)). The difference in current response was calculated for each miRNA-137 concentration and plotted in an excel sheet to calculate the detection limit. As shown in Figure 4(b), the detection limit was calculated as 10 fM with the regression coefficient value of 0.938 on the linear curve, and the sensitivity was found to fall at 1 fM.

### 3.4. Selective and Specific Identification of miRNA-137

**Figure 5:**
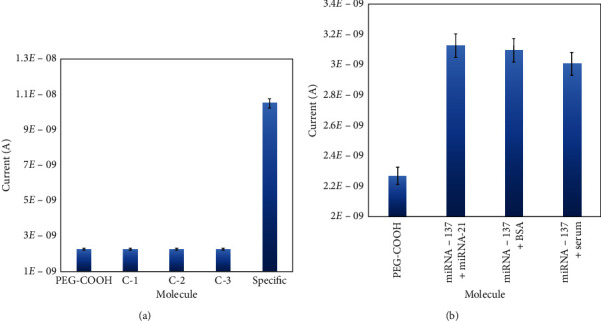
(a) Specific miRNA-137 detection. Experimented with control sequences of miRNA-21 (C-1) and single (C-2) and triple (C-3) base mismatched miRNA-137 sequences. Control sequences did not show any particular current response with complementary miRNA-137 compared with the specific hybridization (S). (b) Specific miRNA-137 detection. Experimented with mixed miRNA-137, miRNA-21, and serum. Mixing other molecules did not interfere the hybridization of miRNA-137 with complementary sequences and confirms the selective detection.

Specific detection of miRNA-137 was confirmed with control sequences of miRNA-21 and single and triple base mismatched miRNA-137 sequences. As shown in Figure 5(a), all three control sequences did not show any particular current response with comp-miRNA-137. At the same time, it shows the clear current response with the specific miRNA-137 sequence. This experiment confirms the specific miRNA-137 hybridization with its complementary sequence. To identify the selective detection, miRNA-137 was mixed with other molecules such as miRNA-21 and diluted human serum. The displayed results from Figure 5(b), mixed miRNA-137 with other biomolecules, did not interfere the hybridization with its complementary sequences, which confirms the selective miRNA-137 detection.  Table 1 is included to display the comparison of the current method to other available strategies. In addition, the advantage of the current method is shown, and these display the potentials of the current sensor.

## 4. Conclusion

Alzheimer's disease (AD) is the neurogenerative disorder, causing cognitive problems and memory loss. It affects everyday lifestyle and makes it difficult to do their other activities individually. Until now, there is no complete cure for AD, but medications improve their quality of life. Diagnosing AD at its earlier stages helps to ensure the proper treatment, so that, it is mandatory for developing a highly sensitive biosensor to detect the AD biomarker. This work was created to determine the AD biomarker miRNA-137 on graphene oxide- (GO-) modified triangular electrodes surface. To improve the sensitivity, comp-miRNA-137 was conjugated with gold nanostar and attached on the GO-modified surface through the amine linker. The detection limit was achieved to 10 fM with the sensitivity of 1 fM, and the control sequences of miRNA-21 and mismatched miRNA-137 sequences failed to hybridize with complementary of miRNA-137, indicating the specific miRNA-137 detection. This research work helps to identify the miRNA-137 sequence and diagnose ideally the AD.

## Figures and Tables

**Table 1:** Comparison among the available methods for Alzheimer's disease identification.

Method	Target	Selectivity	Sensitivity	Advantage	Reference
Electrochemical sensor	MicroRNA	5–750 fM	1.7 fM	Gold-graphene improves detection	[22]
Immunomagnetic biosensor	A*β*42	5–800 pg/mL	5 pg/mL	Cheaper with shorten time	[29]
Electrochemical sensor	A*β*1–42	0.011–55 nM	2.398 pM	Graphene-rGO improves detection	[30]
Immunoelectrochemical sensor	A*β*	0.14–1 ng/mL	0.14 ng/mL	Biocompatible fabrication	[31]
SPR	Acetylcholine	0–10 *μ*M	38 nM	Remote sensing and compactness	[32]
Multiplex aptasensor	A*β*42	0–1000 fM	8.4 fM	Simultaneous detection	[33]
Multiplex aptasensor	Tau-441	0–1000 fM	4.3 fM	Simultaneous detection	[33]
Interdigitated electrode	miRNA-137	1 fM–10 pM	1 fM	Negligible biofouling and improved detection	This work

## Data Availability

The data used to support the findings of this study are included within the article.

## Abbreviations

miRNA-137: MicroRNA-137
AD: Alzheimer's disease
GNS: Gold nanostar
fM: Femtomolar
GO: Graphene oxide
APTMS: 3-Aminopropyltrimethoxysilane
PBS: Phosphate buffer saline
dATP: Deoxyadenosine triphosphate
KOH: Potassium hydroxide
RD: Resist developer
RT: Room temperature
PEG: Polyethylene glycol
pM: Picomolar.
